# Influence of low ambient temperature on epitympanic temperature measurement: a prospective randomized clinical study

**DOI:** 10.1186/s13049-015-0172-5

**Published:** 2015-11-05

**Authors:** Giacomo Strapazzon, Emily Procter, Gabriel Putzer, Giovanni Avancini, Tomas Dal Cappello, Norbert Überbacher, Georg Hofer, Bernhard Rainer, Georg Rammlmair, Hermann Brugger

**Affiliations:** EURAC Institute of Mountain Emergency Medicine, Bolzano, Italy; Department of Anaesthesiology and Critical Care Medicine, Innsbruck Medical University, Innsbruck, Austria; Department of Otolaryngology, General Hospital of Bressanone, Bressanone, Italy; Department of Anesthesiology and Critical Care Medicine, General Hospital of Silandro, Silandro, Italy; Department of Anaesthesiology, Marienklinik, Bolzano, Italy; Department of Anaesthesiology and Critical Care Medicine, General Hospital of Bressanone, Bressanone, Italy

**Keywords:** Epitympanic temperature, Core body temperature, Thermistor thermometer, Non-invasive temperature measurement

## Abstract

**Background:**

Epitympanic temperature (T_ty_) measured with thermistor probes correlates with core body temperature (T_core_), but the reliability of measurements at low ambient temperature is unknown. The aim of this study was to determine if commercially-available thermistor-based T_ty_ reflects T_core_ in low ambient temperature and if T_ty_ is influenced by insulation of the ear.

**Methods:**

Thirty-one participants (two females) were exposed to room (23.2 ± 0.4 °C) and low (−18.7 ± 1.0 °C) ambient temperature for 10 min using a randomized cross-over design. T_ty_ was measured using an epitympanic probe (M1024233, GE Healthcare Finland Oy) and oesophageal temperature (T_es_) with an oesophageal probe (M1024229, GE Healthcare Finland Oy) inserted into the lower third of the oesophagus. Ten participants wore ear protectors (Arton 2200, Emil Lux GmbH & Co. KG, Wermelskirchen, Switzerland) to insulate the ear from ambient air.

**Results:**

During exposure to room temperature, mean T_ty_ increased from 33.4 ± 1.5 to 34.2 ± 0.8 °C without insulation of the ear and from 35.0 ± 0.8 to 35.5 ± 0.7 °C with insulation. During exposure to low ambient temperature, mean T_ty_ decreased from 32.4 ± 1.6 to 28.5 ± 2.0 °C without insulation and from 35.6 ± 0.6 to 35.2 ± 0.9 °C with insulation. The difference between T_ty_ and T_es_ at low ambient temperature was reduced by 82 % (from 7.2 to 1.3 °C) with insulation of the ear.

**Conclusions:**

Epitympanic temperature measurements are influenced by ambient temperature and deviate from T_es_ at room and low ambient temperature. Insulating the ear with ear protectors markedly reduced the difference between T_ty_ and T_es_ and improved the stability of measurements. The use of models to correct T_ty_ may be possible, but results should be validated in larger studies.

## Background

Accurate measurement of core body temperature (T_core_) can be fundamental for guiding treatment and triage decisions in emergency care of patients. Although invasive techniques remain the gold standard for T_core_ measurement (pulmonary artery or lower third of the oesophagus) these sites are not practical in emergency situations [1].

Temperature at the tympanic membrane was originally proposed as a less invasive alternative for estimation of T_core_ [2, 3]. Previous investigations have shown that with precise placement in the lower anterior quadrant of the tympanic membrane, tympanic temperature is highly correlated with intracranial temperature and changes in temperature [4] and, importantly, that tympanic temperature is independent from the influence of changes in skin temperature [3]. Epitympanic temperature (T_ty_) measured with a thermistor probe in the ear canal correlates with T_core_ in hypothermic patients [5, 6] and may be a non-invasive alternative for diagnosing the severity of hypothermia in victims of accidental hypothermia [7, 8]. Case reports of deep hypothermic patients have shown that T_ty_ measured prehospitally was comparable to T_core_ measured invasively at hospital admission [9–11], but there is still a lack of data on the reliability of epitympanic measurements at low ambient temperature. Early studies compared T_ty_ to oesophageal temperature (T_es_) during exposure to moderately low temperatures (between 0 and 10 °C) [3, 12, 13], though ambient conditions in many prehospital situations are commonly much colder with confounding environmental factors. Only two case series (five participants each) describe T_ty_ measured with self-made devices during cold exposure (between −20 and −32 °C) [5, 14]. Pre-hospital use of commercially-available thermometers falls outside the tested operating conditions, since standard probes are validated in-hospital under relatively stable conditions [1]. The aim of this study was to determine if commercially-available thermistor-based T_ty_ reflects T_core_ in low ambient temperature and if T_ty_ is influenced by insulation of the ear.

## Methods

### Design, setting and participants

Volunteers were recruited from the local mountain rescue organization. Written informed consent was obtained from all participants prior to participation in the study. The study was approved by the Ethics Committee of the Regional Hospital of Bolzano, Italy. Participants were in good cardiopulmonary health; a clinical history and medical examination were conducted to exclude acute or chronic conditions or abnormalities of the ear canal or upper airways. Cerumen was removed from the ear canal if necessary. Participants were instructed to fast for at least 6 h prior to testing.

We used a randomized cross-over design. Participants were randomly assigned to group A or B; group A was exposed to low temperature first followed by room temperature (*n* = 15); group B was exposed to room temperature first followed by low temperature (*n* = 16). A climate chamber with controlled temperature settings was used for the low temperature setting and a medical examination office for the room temperature setting.

### Monitoring

An oesophageal probe (9F general purpose sterile probe M1024229, GE Healthcare Finland Oy) was inserted via the naris into the lower third of the oesophagus [15] after anesthesia of the nasal and pharyngeal mucosa with topical 2 % lidocaine solution. An epitympanic probe (M1024233, GE Healthcare Finland Oy) was inserted according to the product instructions into the right ear and fixed to the lobe using standard surgical tape to prevent displacement. Ten participants additionally used industrial ear protectors (Arton 2200, Emil Lux GmbH & Co. KG, Wermelskirchen, Switzerland) to insulate the ear from ambient air. After successful insertion, the probes were connected to an intensive care monitor (Compact Anesthesia Monitor, GE Healthcare Finland Oy).

### Protocol

Probes were placed after at least 30 min of rest. After placement of the probes in the medical examination office, participants were guided to the first test setting. Measurements were recorded every 5 s for a total duration of 10 min for each location (participants were in a seated position for the testing duration). The interval to transfer to the second test setting and commence data recording was between 3 and 5 min. Complete winter clothing including a hat was allowed during measurements at low temperature but was removed during measurements at room temperature.

### Data analysis

Descriptive data are reported as mean ± standard deviation unless otherwise indicated. The Wilcoxon signed-rank test was used to compare (i) T_es_ and T_ty_ in the same person at a specified duration of exposure and (ii) T_es_ or T_ty_ in the same person between the first and last measurement. The Wilcoxon-Mann–Whitney test was used to compare T_es_ or T_ty_ between groups A and B and the Pearson coefficient to correlate differences between T_es_ or T_ty_ in the room and low temperature setting. The Bland-Altman plot and concordance correlation coefficient (CCC) [16] were used to quantify the agreement between T_es_ and T_ty_. A model to correct T_ty_ was developed using a linear regression. The statistical analyses were performed using SPSS software (Version 22.0.0.0, SPSS Inc., Chicago, IL, USA); the Bland-Altman plot and CCC were calculated using MedCalc (Version 9.3.7.0, MedCalc Software, Ostend, Belgium). *P* <0.05 was considered significant.

## Results

There were 31 participants (two females) with mean age 38 ± 12 years (range 22–61 years). The ambient air temperature in the room temperature setting was 23.2 ± 0.4 °C and in the low temperature setting was −18.7 ± 1.0 °C. In one participant the oesophageal probe was displaced after 5 min at room temperature and the last 5 min of measurements had to be excluded from the analysis. In one participant the monitor data was not stored and manually recorded measurements were used for the last 4 min at low temperature.

### Measurements at room temperature

**Fig. 1:**
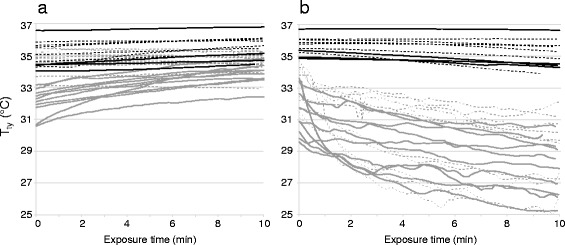
Epitympanic temperature (T_ty_) at room temperature (**a**; 23.2 ± 0.4 °C) and low temperature (**b**; −18.7 ± 1.0 °C) in relation to exposure time in minutes. Data are displayed for group A (solid lines; exposure to low followed by room temperature), group B (dotted lines; exposure to room followed by low temperature), with insulation of the ear (*black*) and without insulation of the ear (*grey*)

Temperature measurements during testing at room temperature are shown in Fig. 1a. Mean T_es_ was 36.8 ± 0.4 °C at 0 min and 36.8 ± 0.3 °C at 9:35 min (*n* = 30, *p* = 0.124). Without insulation of the ear, mean T_ty_ increased from 33.4 ± 1.5 °C at 0 min to 34.2 ± 0.8 °C at 9:35 min (*n* = 21, *p* = 0.004). With insulation of the ear (using the ear protector), mean T_ty_ increased from 35.0 ± 0.8 °C at 0 min to 35.5 ± 0.7 °C at 9:35 min (*n* = 10, *p* = 0.005).

**Fig. 2:**
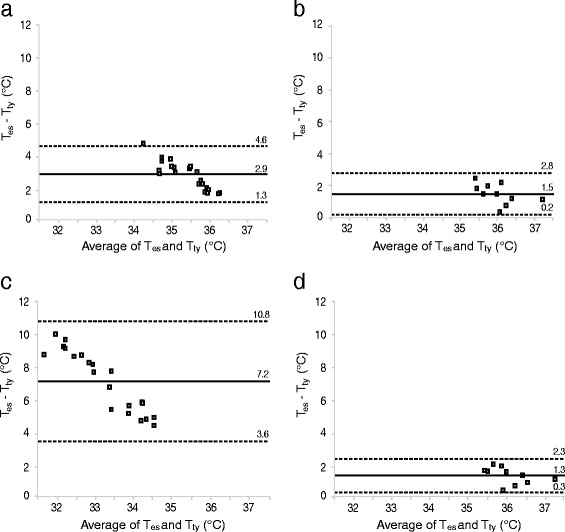
Bland-Altman plots of individual data for mean T_es_ and T_ty_ at room temperature (**a**, without insulation of the ear; **b**, with insulation) and low temperature (**c**, without insulation of the ear; **d**, with insulation). The solid line is the mean of differences and the dashed lines are the limits of agreement (mean ± 1.96 standard deviation)

Based on a Bland-Altman plot (Fig. 2), the mean difference between T_ty_ without insulation of the ear and T_es_ was 2.9 °C and the correlation was weak (CCC = 0.03, 95 % confidence interval [CI] 0.00–0.06, *n* = 21). The mean difference was less with insulation of the ear (1.5 °C) and the correlation was not significant (CCC = 0.11, 95 % CI −0.05–0.27, *n* = 10).

In the room temperature setting, T_ty_ without insulation of the ear was lower at 0 min in group A (32.0 ± 0.9 °C; *n* = 10) compared to B (34.6 ± 0.7 °C; *n* = 11, *p* <0.001), but was not different with insulation of the ear (*p* = 0.476).

### Measurements at low temperature

Temperature measurements during testing at low temperature are shown in Fig. 1b. Mean T_es_ was 36.7 ± 0.4 °C at 0 min and 36.8 ± 0.4 °C at 9:15 min (*n* = 31, *p* = 0.001). Without insulation of the ear, mean T_ty_ decreased from 32.4 ± 1.6 °C at 0 min to 28.5 ± 2.0 °C at 9:15 min (*n* = 21, *p* <0.001). With insulation of the ear, mean T_ty_ decreased from 35.6 ± 0.6 °C at 0 min to 35.2 ± 0.9 °C at 9:15 min (*n* = 10, *p* = 0.007).

Based on a Bland-Altman plot (Fig. 2), the mean difference between T_ty_ without insulation of the ear and T_es_ was 7.2 °C and the correlation was not significant (CCC = 0, 95 % CI −0.01–0.01, *n* = 21). The mean difference was less with insulation of the ear (1.3 °C), which was similar to the mean difference at room temperature (1.5 °C). The correlation between T_ty_ with insulation of the ear and T_es_ was not significant (CCC = 0.18, 95 % CI −0.02–0.37, *n* = 10).

In the low temperature setting, T_ty_ without insulation of the ear was lower at 0 min in group A (31.5 ± 1.4 °C; *n* = 10) compared to B (33.3 ± 1.3 °C; *n* = 11, *p* = 0.008), but was not different with insulation of the ear (*p* = 0.352).

### Individual variability in temperature

To understand the influence of inter-individual variability, the difference between T_ty_ and T_es_ at 3 min at room temperature was correlated to the difference between T_ty_ and T_es_ at 3 min at low temperature for each individual. We chose 3 min to reduce the variability seen in some measurements in the first minutes after exposure. The measurements were correlated, suggesting that individuals with a large deviation between T_ty_ and T_es_ in one setting also had a large deviation in the other setting. The correlation was stronger with insulation of the ear (*r* = 0.907, *n* = 10, *p* <0.001) compared to without (*r* = 0.446, *n* = 21, *p* = 0.043).

### Model for predicting core temperature

**Fig. 3:**
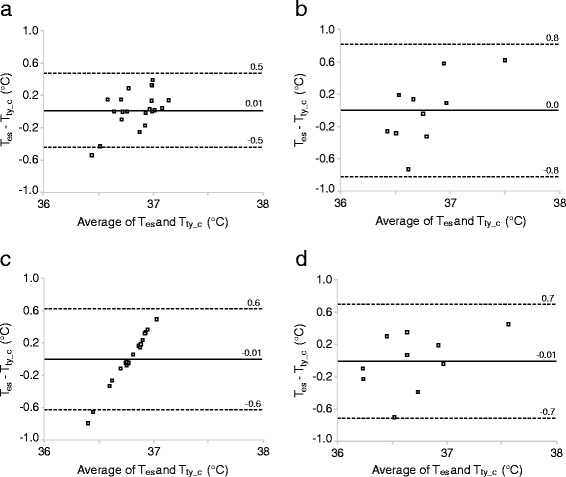
Bland-Altman plots of individual data for T_es_ and T_ty_c_ at room temperature (**a**, without insulation of the ear; **b**, with insulation) and low temperature (**c**, without insulation of the ear; **d**, with insulation). The solid line is the mean of differences and the dashed lines are the limits of agreement (mean ± 1.96 standard deviation). T_ty_c_ are corrected values of T_ty_ at 3 min derived from a linear regression model to predict T_es_

T_ty_ was corrected (T_ty_c_) using a linear regression model to predict T_es_ from T_ty_ at 3 min . The model at room temperature was *T*_*ty_c*_ = 32.32 + 0.134 * T_*ty*_ without insulation of the ear and *T*_*ty_c*_ = 26.394 + 0.295 * T_*ty*_ with insulation. The model at low temperature was *T*_*ty_c*_ = 37.025 - 0.008 * T_*ty*_ without insulation and *T*_*ty_c*_ = 17.15 + 0.55 * T_*ty*_ with insulation. Based on a Bland-Altman plot (Fig. 3), the differences between T_ty_c_ and T_es_ were correlated at room temperature without insulation (CCC = 0.446, 95 % CI 0.129–0.681, *n* = 21) and at low temperature with insulation (CCC = 0.659, 95 % CI 0.172–0.887, *n* = 10); at room temperature with insulation the correlation was not significant (CCC = 0.389, 95 % CI −0.107–0.730, *n* = 10). At low temperature without insulation, Fig. 3c shows that model correction was not effective and measurements were not correlated (CCC = 0.009, 95 % CI −0.036–0.054, *n* = 21).

## Discussion

We report the influence of low ambient temperature and individual factors on temperature measured epitympanically using a thermistor-based probe. We found a deviation from the reference T_core_ that was markedly reduced with insulation of the ear from ambient air using an ear protector. We also propose a model to correct T_ty_. Nevertheless, inter-individual variability remained and variations in probe placement likely account for some of the variability.

### Influence of ambient temperature

Epitympanic measurements were influenced by ambient temperature. During exposure to room temperature, mean T_ty_ increased by 0.8 °C, whereas it decreased by 3.9 °C during exposure to low temperature. Similarly, mean deviation from T_es_ was >50 % higher in the low temperature setting compared to the room temperature setting. However, it is interesting that the difference to T_es_ in our data was reduced with simple external insulation with an ear protector; with insulation the mean difference to T_es_ was reduced by 52 % in the room temperature setting (difference of 2.9 to 1.5 °C) and by 82 % in the low temperature setting (7.2 to 1.3 °C; Fig. 2). This suggests that the ear cover limits exchange of ambient air, thus creating a “microclimate” in the ear canal, though the influence of ambient temperature on T_ty_ was not completely removed. Moreover, previous studies of T_ty_ that also insulated the ear or ear canal reported that measurements required several minutes to stabilize [5, 12, 13], even if an external device was used to heat the ear protector [12]. In our study, T_ty_ without insulation of the ear did not stabilize and showed rapid and constant decreases over 10 min in low ambient temperature. This explains the differences in T_ty_ at baseline between group A and B—for example, in the room temperature setting mean T_ty_ at 0 min was lower in group A than B because they had already been exposed to low ambient temperature and adaptation to the warmer temperature was still occurring in the first minutes.

### Inter-individual variability

We found that the degree of deviation of T_ty_ from T_es_ in both ambient air settings was different between individuals, i.e. individuals with a large deviation in one setting also had a large deviation in the other setting. This is partially due to differences in placement of the probe. Temperature in the ear canal seems to decrease with increasing distance from the tympanic membrane [17, 18], and thus T_ty_ will be less reflective of T_core_ with increasing distance. The distance between the sensor and the tympanic membrane will vary slightly between individuals because of differences in anatomy (length, width, shape) that affect insertion depth. Additionally, there may be other unknown physiological factors such as individual differences in the vascular anatomy of the ear and thermal conductivity and perfusion of the tissues that could influence the absolute difference between T_ty_ and T_core_ in an individual.

### Practical implications

Measuring T_core_ is the only way to accurately assess the severity of hypothermia. There are other commonly used scales to stage hypothermia based on clinical signs and symptoms, but these are not always reliable since there are differences in consciousness among patients at a given T_core_ [19]. Patients with mild hypothermia (T_core_ 35 to 32 °C) can be treated in the field if they are uninjured or transported to the closest hospital if in-field rewarming is not possible, whereas patients with moderate hypothermia (T_core_ <32 °C) should be transported to the most appropriate hospital on the basis of cardiac stability [7]. The results of our study show a large difference (7.2 °C) between mean T_ty_ and T_core_ at low temperature, which in practical terms could lead to wrong triage and transport even of normothermic patients. These results also suggest that T_ty_ without insulation of the ear does not stabilize within 10 min, and thus is impractical for applications that require rapid measurement and/or accurate monitoring of changes in T_core_.

The models to correct T_ty_ seem to give a valid estimation of T_es_ for measurements at room temperature without insulation of the ear and at low temperature with insulation. It was unexpected that the correlation between T_ty_c_ and T_es_ at room temperature with insulation was low and non-significant, though this is probably due to the small sample size (*n* = 10). In comparison to these three conditions, it was not possible to create a realistic model for measurements at low temperature without insulation. Thus in order to reliably predict T_core_ from epitympanic temperature, modification of currently available devices (i.e. ear cover) and development of an appropriate model to correct for ambient temperature may be necessary. Thermistor-based T_ty_ probes are non-invasive and easy to use and could be used with standard patient monitors. This would also allow continuous monitoring of ECG and T_core_, as recommended in international guidelines for pre-hospital management of accidental and therapeutic hypothermia [7].

### Limitations and further research

The results are based on measurements in healthy, normothermic volunteers at rest in the two reported ambient temperatures and may not be applicable for other populations (e.g. patients in cardiac arrest, trauma) or ambient conditions. The models to correct T_ty_ should be validated with a larger sample size. The lack of correlation between T_es_ and T_ty_ (or T_ty_c_) at room temperature with insulation may be due to the small sample size (*n* = 10). Further investigations are needed to understand if models could account for other factors characteristic of the prehospital environment such as rapidly changing conditions and wind.

## Conclusion

Epitympanic temperature measurements are influenced by ambient temperature and deviate from T_es_ at room temperature and low ambient temperature. Insulating the ear with standard industrial ear protectors markedly reduced the difference between T_ty_ and T_es_ and improved the stability of measurements over the testing duration. The use of models to correct T_ty_ may be possible, but results should be validated in larger studies.

### Ethics, consent and permissions

The study was approved by the Ethics Committee of the Regional Hospital of Bolzano, Italy. Written informed consent was obtained from all participants prior to participation in the study.
